# Niclosamide—A promising treatment for COVID‐19

**DOI:** 10.1111/bph.15843

**Published:** 2022-04-11

**Authors:** Shivani Singh, Anne Weiss, James Goodman, Marie Fisk, Spoorthy Kulkarni, Ing Lu, Joanna Gray, Rona Smith, Morten Sommer, Joseph Cheriyan

**Affiliations:** ^1^ Division of Pulmonary and Critical Care Medicine NYU School of Medicine New York New York USA; ^2^ Novo Nordisk Foundation Center for Biosustainability Technical University of Denmark Kongens Lyngby Denmark; ^3^ UNION Therapeutics Research Services Hellerup Denmark; ^4^ Department of Medicine Cambridge University Hospitals NHS Foundation Trust Cambridge UK; ^5^ Cambridge Clinical Trials Unit Cambridge University Hospitals NHS Foundation Trust Cambridge UK; ^6^ UNION Therapeutics Hellerup Denmark

**Keywords:** clinical trials, COVID‐19, niclosamide, repurposing

## Abstract

Vaccines have reduced the transmission and severity of COVID‐19, but there remains a paucity of efficacious treatment for drug‐resistant strains and more susceptible individuals, particularly those who mount a suboptimal vaccine response, either due to underlying health conditions or concomitant therapies. Repurposing existing drugs is a timely, safe and scientifically robust method for treating pandemics, such as COVID‐19. Here, we review the pharmacology and scientific rationale for repurposing niclosamide, an anti‐helminth already in human use as a treatment for COVID‐19. In addition, its potent antiviral activity, niclosamide has shown pleiotropic anti‐inflammatory, antibacterial, bronchodilatory and anticancer effects in numerous preclinical and early clinical studies. The advantages and rationale for nebulized and intranasal formulations of niclosamide, which target the site of the primary infection in COVID‐19, are reviewed. Finally, we give an overview of ongoing clinical trials investigating niclosamide as a promising candidate against SARS‐CoV‐2.

**Abbreviations**
*C*_max_
: maximum concentration
CoV: coronavirus
FDA: Food and Drug Administration
MRSA: Methicillin‐resistant *Staphylococcus aureus*
mTORC: mechanistic target of rapamycin complex
NCT: National ClinicalTrials.gov number
SARS‐CoV‐2: severe acute respiratory syndrome coronavirus‐2
SoC: standard of care

## 1 INTRODUCTION

The outbreak of coronavirus disease 2019 (COVID‐19) caused by the severe acute respiratory syndrome coronavirus 2 (SARS‐CoV‐2) was declared a pandemic by the World Health Organization (WHO) on 11 March 2020. A rapid rise in the numbers of COVID‐19 cases followed (Paules et al., 2020) and as of January 2022, there have been over 296 million cases of COVID‐19 and 5.5 million deaths (WHO, 2022). Healthcare systems have been globally overwhelmed (Miller et al., 2020) and projection studies predict that rapid transmission dynamics will potentially be at play well into 2025 (Kissler et al., 2020). Viral vector and mRNA vaccines have proved an overwhelming success in controlling the pandemic (Rotshild et al., 2021) and as of January 2022, nearly 9 billion vaccine doses have been administered (WHO, 2022). However, vaccines alone are unlikely to be sufficient in vulnerable patient populations (i.e. elderly, cancer, primary immune deficiencies, transplant recipients or dialysis) and patients who receive concurrent medical treatments that supress the immune system may not mount a satisfactory antibody response. Additionally, global herd immunity is unlikely to be achieved due to the constant evolution of new variants of concern and waning of natural or stimulated antibody responses over time. Geopolitical logistics and vaccine nationalism also make it unlikely that these vaccines will be equitably available across the globe for a few more years. Therefore, parallel strategies such as effective novel therapeutic interventions must be rapidly developed. Intensive and collaborative research efforts have resulted in a number of treatment options for COVID‐19. The antivirals, remdesivir (Beigel et al., 2020) and molnupiravir (Jayk Bernal et al., 2021), neutralizing monoclonal antibodies, casirivimab, imdevimab (Somersan‐Karakaya et al., 2021) and sotrovimab (Gupta et al., 2021), IL‐6 antibodies, tocilizumab (Gordon et al., 2021; RECOVERY Collaborative Group, 2021) and dexamethasone (Horby et al., 2021) are the primary treatment options currently approved for COVID‐19, but they can result in serious systemic adverse effects and only moderately affect the clinical outcomes. The limitations of these therapies highlight the need for ongoing development of life‐saving medications to help fight the virus.

Coronaviruses (CoVs) are large, enveloped, positive sense and single‐stranded RNA viruses belonging to the family *Coronaviridae* within the order *Nidovirales* (Y. Chen et al., 2020). They can infect several mammalian hosts and are divided into four genera: ‐ Alpha, Beta, Gamma and Delta, of which Alpha and Beta CoVs are known to infect humans. Full‐genome sequencing and phylogenetic analyses have indicated that the CoV that causes COVID‐19 was in the same subgenus as the SARS virus (Fehr & Perlman, 2015) and was named on the basis of its appearance under electron microscopy. Human CoV infections usually cause mild, self‐limiting respiratory infection. However, the epidemics of SARS‐CoV and Middle East respiratory syndrome coronaviruses (MERS‐CoV) caused alarming morbidity and mortality in 2002–2003 and 2012, respectively (Gao et al., 2016), and COVID‐19 has underscored the continued risk of pandemics caused by such viruses. Risk factors for severe COVID‐19 across the globe include old age, race, gender, obesity, cardiovascular disease, diabetes, chronic lung disease and immunosuppression. Therefore, drugs that target pleiotropic mechanisms may be important. CoVs have a large genome and a higher mutation rate compared with other RNA viruses, hence eradicating them definitively is difficult (Gralinski & Baric, 2015). Broad‐spectrum inhibitors of emerging CoVs are therefore needed and repurposing existing drugs has been validated as a means to tackle the SARS‐CoV‐2 pandemic, as well as enabling future pandemic preparedness.

## 2 REPURPOSING OLD DRUGS FOR A NEW CAUSE

Drug repurposing has emerged as an attractive alternative to the conventional approach of drug discovery, which is often exhaustive and arduous (Ashburn & Thor, 2004). It is a process of identifying new therapeutic roles for a drug that has already been established for the treatment of another condition. The discovery of a new drug and its journey to the market is a process fraught with risks involving toxicity and lack of efficacy, costing billions of dollars and requiring a long timeline. Repurposing therefore offers several advantages over *de novo* drug development, such as reduced development timelines, reduced costs and substantially lower risks, as the safety and pharmacokinetic profile of the drug is already established (S. Pushpakom et al., 2019). The risk of failure is lower because the repurposed drug has been shown to be safe in preclinical models and humans, provided early‐stage trials have been completed. As a result, the timeframe for drug development is significantly shorter (Breckenridge & Jacob, 2019).

Historically, drug repurposing has been mostly serendipitous, usually after a drug was found to have a newly recognized off‐target effect (Nosengo, 2016). Well‐known examples are the use of minoxidil for hair loss, sildenafil for erectile dysfunction and thalidomide for multiple myeloma (Pushpakom et al., 2019). However, recent successes have encouraged the development of more systematic approaches resulting in the identification of a number of promising candidate drugs (Hurle et al., 2013). In more recent years, drug repurposing screens have emerged as an attractive strategy to respond swiftly to emerging infectious diseases (Ashburn & Thor, 2004). Food and Drug Administration (FDA; U.S.A.)‐approved drugs that can achieve a modest antimicrobial activity are a safe and increasingly popular response mechanism to emerging infections. The drugs concerned can be made immediately available for use in clinical trials as they have known safety profiles at the licensed doses and this has had a huge impact during the COVID‐19 pandemic. For example, dexamethasone was repurposed in the RECOVERY trial (Horby et al., 2021) and is estimated to have saved 1 million lives globally by March 2021 (NHS England, 2021) whereas tocilizumab, a repurposed drug for moderate to severe rheumatoid arthritis, is licensed for treating hospitalized patients with moderate to severe COVID‐19 (Gordon et al., 2021; RECOVERY Collaborative Group, 2021). The CORONA Project (a joint initiative by the Castleman Disease Collaborative Network and the Centre for Cytokine Storm Treatment & Laboratory) is tracking all other novel and repurposed drugs for COVID‐19 and assigns the drugs a ‘grade’, based on treatment efficacy and whether pre‐specified endpoints are met (Venkatesan, 2021).

Here, we review the preclinical models that have demonstrated the pluripotential effects of niclosamide as well as some limited human data in a variety of disease states that suggest it could be an agent of choice in COVID‐19.

## 3 HISTORY AND MECHANISM OF ACTION OF NICLOSAMIDE

**FIGURE 1:**
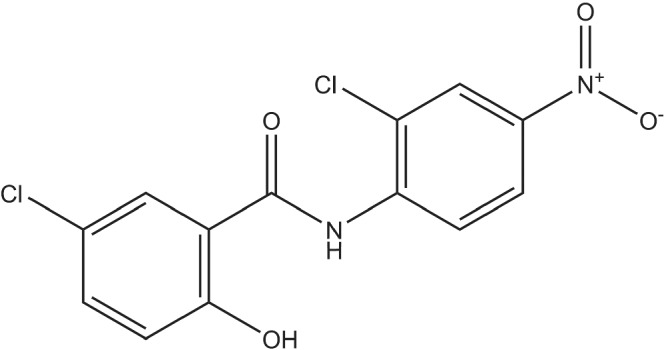
The chemical structure of niclosamide, C_13_H_8_Cl_2_N_2_O_4_

Structurally, niclosamide (Figure 1) belongs to a large group of lipophilic, weakly acidic molecules called salicylanilides, a derivative of salicylic acid (Pearson & Hewlett, 1985). Niclosamide is listed on the WHO's list of essential medications and chewable tablets have been approved for use as an anti‐helminthic agent for cestode (tapeworm) infections for over 40 years with a daily oral dose of 2 g niclosamide (Andrews et al., 1982). Niclosamide inhibits oxidative phosphorylation and stimulates ATP activity in the mitochondria of cestodes, killing both the scolex and the proximal segments of the tapeworm (Al‐Hadiya, 2005; Weinbach & Garbus, 1969). It has been shown to affect several signal transduction pathways such as Wnt/β‐catenin, mechanistic target of rapamycin (mTOR) complex 1 (mTORC1), signal transducer and activator of transcription 3 (STAT3), nuclear factor kappa‐light‐chain‐enhancer of activated B cells (nuclear factor‐κB; NF‐κB) and Notch pathways, all indicating its potential to treat conditions such as cancer (summarized in Table 1), chronic medical diseases, and bacterial and viral infections.

**TABLE 1:** Mechanism of action of niclosamide in various cancers

Cancer type	Mechanism of action	Overall effect	Reference
Colorectal cancer	Interference with the nuclear β‐catenin‐Bcl9‐LEF/TCF triple‐complex and up‐regulation of c‐jun leading to reduced Wnt activity	Inhibitory effect on CRC development	Monin et al. (2016)
	Co‐localization of Frizzled 1 or β‐catenin with LC3, (an autophagosome marker), inhibition of mTORC1 and ULK1	Suppression of CRC via autophagy	J. Wang et al. (2019)
	Down‐regulation of Notch signalling and up‐regulation of tumour suppressor microRNAs (miR‐200)	Suppression of growth and migration of colon cancer cells, and induction of cell apoptosis	Suliman et al. (2016)
	Inhibition of the STAT3 pathway via down‐regulation of survivin and cyclin‐D1	Induction of cell apoptosis and cell cycle arrest	M. M. Wu et al. (2020)
Lung cancer	Caspase‐3 activation and decreased expression level of c‐myc protein	Synergistic effect in cisplatin‐resistant cells and induction of apoptosis	Zuo et al. (2018)
	Blockage of p‐STAT3 binding to the PD‐L1 promoter leading to T‐cell‐mediated cell lysis	Delayed tumour growth in presence of PD‐L1 antibody	Luo et al. (2019)
	Inhibition of S100A4, reduced NF‐κB‐mediated MMP‐9 expression	Reduction in proliferation and invasion of cells	Stewart et al. (2016)
Breast cancer	Inhibition of IL‐6/STAT3 and down‐regulation of TWIST and SNAIL	Inhibition of adipocyte‐induced epithelial mesenchymal transition leading to decreased proliferation, migration and invasion of human breast cancer cell lines and induction of apoptosis	Gyamfi et al. (2019)
	Activation of caspase‐3 and down‐regulation of Bcl‐2, Mcl‐1 and survivin	Growth inhibition of breast cancer cell lines and induction of apoptosis	Ye et al. (2014)
Head and neck cancer	Inhibition of STAT3	Apoptosis in tumour cells and growth suppression. Synergistic effect with EGFR inhibition	R. Li et al. (2013)
	Increased let‐7d expression and decreased CDC34 expression	G1 arrest in squamous cell carcinoma cell lines and inhibition of xenograft growth	Han et al. (2018)
	Down‐regulation of VEGFA, MMP2, ROCK1 and CdC42. Down‐regulation of phosphorylated STAT3 expression and up‐regulation of miR‐124	Inhibition of proliferation, promotion of apoptosis, inhibition of vasculogenic mimicry *in vitro* and *in vivo*	X. Li et al. (2018)
	Inhibition of Wnt/β‐catenin signalling pathway, down‐regulation of glycogen synthase kinase‐3β and cyclin D1	Reduced formation of primary and secondary tumourspheres and inhibition of EMT in oral squamous cell carcinoma cells	L. H. Wang, Xu, et al. (2018)
Ovarian cancer	Decreased expression of p‐STAT3, inactivation of MEK1/2‐ERK1/2 pathways and decreased mitochondrial respiration and aerobic glycolysis	Suppression of ovarian carcinoma growth and induction of cell apoptosis	Shangguan et al. (2020)
	Decreased expression of proteins in the Wnt/β‐catenin, mTOR and STAT3 pathways and preferential targeting of CD133	Inhibition of chemo‐resistant ovarian cancer cells	Arend et al. (2016)
	Inhibition of Wnt/β‐catenin pathway	Decreased cellular proliferation and increased cell death	Arend et al. (2014)
Glioblastoma multiforme	Inhibition of WNT, NOTCH, mTOR and NF‐κB signalling cascades	Cytostatic, cytotoxic and anti‐migratory effect on human glioblastoma cell lines and diminution of its malignant potential *in vivo*	Wieland et al. (2013)
	Down‐regulation of Wnt/β‐catenin, PI3K/AKT, MAP kinase and STAT3	Increase in protein ubiquitination and autophagy, leading to induction of toxicity in human glioblastoma cells	Cheng et al. (2017)
Hepatocellular carcinoma	Down‐regulation of expression of cyclin D1 and MMP‐9	Suppressed proliferation and migration of HCC cells lines	Tomizawa et al. (2015)
	Down‐regulation of cyclin D1 via a down‐regulation of the Wnt‐3 pathway	Suppressed proliferation of HCC cells by induction of apoptosis	Tomizawa et al. (2013)
	Inhibition of STAT3 and more downstream anti‐apoptotic proteins	Suppressed cell viability and inhibited clone formation in HCC cells and synergism with cisplatin by promotion of apoptosis	Wang, Zhou, et al. (2018)
	Reduced expression of proteins in the Wnt‐β–catenin, STAT3, AKT–mTOR and epidermal growth factor signalling pathways	Increased expression of 20 genes that are down‐regulated and reduced expression of 29 genes that are up‐regulated in the 274‐gene HCC signature	B. Chen et al. (2017)
Leukaemia	Increased levels of reactive oxygen species, inhibition of glutathione synthesis and NFAT signalling	Anti‐tumour activity seen against drug‐resistant leukaemia cells	Hamdoun et al. (2017)
	Inhibition of NF‐κB	Synergism with frontline chemotherapeutic agents in killing acute myeloid leukaemia cells	Y. Jin et al. (2010)
Prostate cancer	Inhibition of STAT3 phosphorylation via IL‐6 pathway	Induction of apoptosis and inhibition of cell growth and cell migration in enzalutamide‐resistant prostate cancer cells	Liu et al. (2015)
	Inhibition of androgen receptor variant 7	Reversal of enzalutamide and bicalutamide resistance in castrate‐resistant prostate cancer cells	Liu et al. (2017)

Abbreviations: Bcl, B‐cell lymphoma; CRC, colorectal cancer; EGFR, epidermal growth factor receptor; EMT, epithelial to mesenchymal transition; ERK, extracellular signal‐regulated protein kinase; HCC, hepatocellular carcinoma; LC3, microtubule‐associated protein 1A/1B‐light chain 3; LEF, lymphoid enhancer factor; MAP, mitogen‐activated protein; MCL‐1, myeloid cell leukaemia sequence 1; MEK, mitogen‐activated protein kinase kinase; MMP, matrix metallopeptidase; mTOR, mechanistic target of rapamycin; NFAT, nuclear factor of activated T cells; NF‐κB, nuclear factor kappa‐light‐chain‐enhancer of activated B cells; PD‐L1, programmed death‐ligand 1; PI3K, phosphoinositide 3‐kinases; ROCK, rho‐associated coiled‐coil kinase; STAT3, signal transducer and activator of transcription 3; TCF, T‐cell factor; VEGF, vascular endothelial growth factor.

Oral niclosamide is only partially absorbed from the intestinal tract with a low bioavailability of 5.5%–10% and is rapidly eliminated by the kidneys with no cumulative toxic effects (Andrews et al., 1982). Distribution studies in rodents with oral doses of 40 mg·kg^−1^ niclosamide ethanolamine (i.e. ~34 mg·kg^−1^ niclosamide) and 50 mg·kg^−1^ niclosamide demonstrate that tissue levels are highest in excretory organs (intestines, liver and kidney) and low to negligible levels are achieved in other tissues (such as the brain, heart and lungs) (Duhm et al., 1961; Tao et al., 2014).

### 3.1 Pharmacokinetics of oral niclosamide

Oral administration of niclosamide in rodents reached a mean maximum plasma concentration (*C*_max_) of 22.4 ng·ml^−1^ (1 mg·kg^−1^), 354 ng·ml^−1^ (5 mg·kg^−1^) and 644 ng·ml^−1^ (~34 mg·kg^−1^) (Chang et al., 2006; Choi et al., 2021; Tao et al., 2014). In humans receiving niclosamide (2 g·day^−1^, p.o.), the median *C*_max_ ranged from 665 to 759 ng·ml^−1^ in five colorectal cancer patients and from 250 to 6000 ng·ml^−1^ in healthy subjects (Andrews et al., 1982; Burock, Daum, Keilholz, et al., 2018). In three prostate cancer patients receiving 1.5 g·day^−1^, p.o., the *C*_max_ ranged from 35.7 to 182 ng·ml^−1^, respectively (Schweizer et al., 2018a, 2018b).

Therapeutic plasma levels in rodent studies (using doses from 5 mg·kg^−1^·day^−1^, p.o., to 200 mg·kg^−1^·day^−1^, p.o.) likely exceed the plasma levels in humans prescribed a 2 g p.o. dose, based on the results from Tao et al. (2014) and assuming dose linearity. This therefore raises additional pharmacokinetic (PK) and pharmacodynamic (PD) challenges as well as potential toxicity issues for human clinical trials.

## 4 NICLOSAMIDE IN CHRONIC MEDICAL STATES

### 4.1 Niclosamide and metabolic syndrome

A seminal study from Tao et al. (2014) showed that niclosamide ethanolamine (150 mg·kg^−1^·day^−1^, p.o.) reduced liver fat accumulation (steatosis) in mice fed a high‐fat diet. The effects were also studied in human liver cells and demonstrated increased lipid oxidation and up‐regulation of the AMP‐activated protein kinase pathway, suggesting its potential use as an anti‐obesity agent. In a murine obesity study, mice were fed either a chow or high‐fat diet and niclosamide (150 mg·kg^−1^·day^−1^, p.o., or 75 mg·kg^−1^·day^−1^, p.o.) and niclosamide ethanolamine for 10 weeks (Chowdhury et al., 2017). Both niclosamide and niclosamide ethanolamine significantly improved glucose metabolism by inhibiting the glucagon signalling pathway, without altering total body weight or insulin secretion or sensitivity. In a further study, administration of niclosamide (140 mg·kg^−1^·day^−1^, mixed in food) to high‐fat‐diet‐fed mice, resembling a murine obesity model, for 4 weeks resulted in a significant decline in food intake and body weight compared with control mice (Al‐Gareeb et al., 2017). There was also significant lowering of the fasting blood glucose, fasting plasma insulin and improved insulin resistance.

### 4.2 Niclosamide and autoimmunity

In a collagen‐induced arthritis rat model, niclosamide (100 mg·kg^−1^, p.o.) significantly reduced the arthritis index, footpad thickness and ankle swelling (Al‐Gareeb et al., 2019). Histopathological examination revealed reduced infiltration of inflammatory cells along with reduced bone and cartilage destruction. Serum levels of tumour necrosis factor α (TNFα) and IL‐1β were also significantly reduced compared with control animals. In a murine lupus study using MRL/*lpr* mice, daily administration of niclosamide (100 mg·kg^−1^, oral gavage) for 7 weeks improved proteinuria, anti‐dsDNA antibody levels, histological features and ameliorated C3 deposition in the kidneys (Jang et al., 2021). Niclosamide also led to a reduction of splenic follicular helper T cells and plasma cells STAT3 inhibition. Finally, in a hypochlorous acid‐induced mouse model of systemic sclerosis, niclosamide (10 mg·kg^−1^ 5 days·week^−1^ for 6 weeks, i.p.) reduced clinical markers of skin fibrosis, such as skin thickening and collagen content, as well as skin levels of IL‐4 and IL‐13. There was a decrease in STAT3, AKT and Wnt/β‐catenin pathways in the skin of hypochlorous acid‐induced mice (Morin et al., 2016). Finally, in an Iraqi study, patients with active rheumatoid arthritis on etanercept showed a good response to adjuvant niclosamide therapy (1 g·day^−1^, p.o.). Significant improvements in joint and clinical severity indices and a decrease in the serum levels of IL‐1β, E‐selectin, intercellular adhesion molecule 1 (ICAM1) and vascular cell adhesion protein 1 (VCAM1) were observed (Al‐Gareeb et al., 2018).

### 4.3 Niclosamide and pulmonary pathology

In a screen of ∼580,000 compounds, niclosamide was identified as a TMEM16A antagonist, a calcium‐activated chloride channel (CaCC) that contributes to mucus hypersecretion and bronchoconstriction in reactive airway disease (Miner et al., 2019). The study tested efficacy, using maximally contracted and cytokine‐treated airways,. and confirmed that niclosamide had a potent bronchodilator effect. In transgenic asthmatic mice, niclosamide (13 mg·kg^−1^·day^−1^ for 3 days, i.p.) reduced mucus production and bronchoconstriction, and also demonstrated anti‐inflammatory and antibacterial effects (Cabrita et al., 2019). Centeio et al. (2021) further investigated these findings, demonstrating that intratracheal instillation of 0.98% niclosamide for 4 days inhibited mucus production and secretion in ovalbumin‐sensitized mice. Niclosamide has been found to exert anti‐fibrotic effects via Wnt/β‐catenin signalling in a cellular model as well as in a bleomycin‐induced murine pulmonary fibrosis model, following once‐daily administration of low‐dose niclosamide (3 mg·kg^−1^, i.p.) and high‐dose niclosamide (10 mg·kg^−1^, i.p.) for 21 days (Boyapally et al., 2019). In rats with established monocrotaline‐induced pulmonary hypertension, niclosamide (75 mg·kg^−1^·day^−1^ for 14 days, p.o.) reduced vascular remodelling and improved right heart function via STAT3 inhibition (Braga et al., 2020). There was reduced expression of TGF‐β, hypoxia‐inducible factor 1α (HIF) and vimentin, a mesenchymal marker, along with reduced epithelial to mesenchymal transition (EMT).

### 4.4 Niclosamide and cancer

Several studies have demonstrated that niclosamide has potent *in vitro* and *in vivo* effects against a wide range of cancers. We have further summarized animal and preclinical studies that highlight the potential use of niclosamide in cancer in Table 1. In a Phase 1 dose‐escalation study testing oral niclosamide plus standard dose enzalutamide for prostate cancer conducted by Schweizer et al. (2018a, 2018b), subjects on the higher dose (1000 mg three times per day, p.o.) experienced dose‐limiting toxicities including vomiting, diarrhoea and colitis. Plasma concentrations at this maximum dose were below the therapeutic threshold and therefore, the study was closed due to ineffectiveness. A further, more recent Phase Ib trial identified the maximum tolerated dose of a novel reformulated version of niclosamide and recommended a Phase 2 study of this orally bioavailable form in combination with abiraterone and prednisolone in men with castration‐resistant prostate cancer (Parikh et al., 2021). Nine patients were recruited and no dose‐limiting toxicity was observed at any dose. Five out of eight patients achieved a prostate‐specific antigen response, two achieved undetectable prostate‐specific antigen levels and a radiographic response. The NIKOLO clinical trial is a Phase II, single‐centre, one‐arm open‐label study to investigate the safety and efficacy of niclosamide in patients with metastatic colorectal cancer progressing under standard therapy (Burock, Daum, Keilholz, et al., 2018; Burock, Daum, Tröger, et al., 2018). The study is focused on the feasibility, toxicity and efficacy of niclosamide in order to explore new treatment options. It is worth noting that despite ongoing trials, niclosamide is not approved for the treatment of any oncological condition.

### 4.5 Niclosamide and other models of disease

In a murine endometriosis model, established by surgically inducing endometriotic‐like lesions, niclosamide (100 and 200 mg·kg^−1^, p.o.) decreased the growth rate and progression of endometriosis‐like lesions and inhibited STAT3 and NF‐κB pathways (Prather et al., 2016). Inhibition of macrophage‐induced inflammatory pathways has been observed in primary endometriotic stromal cells (Sekulovski et al., 2020). Niclosamide has also been shown to act as a potential neuroprotective agent such as in Parkinson's disease by activating PINK1 in HeLA and primary cortical neurons (Barini et al., 2018), reduce neuro‐inflammation in activated murine primary microglia *in vitro* (Serrano et al., 2019) and block signalling pathways associated with neuropathic pain in rats receiving 1 mM niclosamide intratracheally (Zhang et al., 2013).

## 5 NICLOSAMIDE AS AN ANTIBACTERIAL AGENT

Niclosamide has well‐identified antimicrobial properties and has been shown to prevent the formation of biofilms of hospital‐acquired and device‐associated gram‐positive bacteria, such as *Staphylococcus aureus* and methicillin‐resistant *S. aureus* (MRSA) at concentrations as low as 0.01 μg·ml^−1^ (Gwisai et al., 2017). When applied as a device coating, niclosamide prevented bacterial attachment and demonstrated potent antimicrobial activity (Gwisai et al., 2017). In a screen of 1280 commercially available drugs, niclosamide was one of nine agents that possessed antimicrobial activity against preformed biofilms (Torres et al., 2016). A time‐kill study further showed that niclosamide is bacteriostatic against a number of gram‐positive bacteria including MRSA, displaying strong *in vivo* (*Caenorhabditis elegans*‐MRSA liquid infection model) as well as *in vitro* activity (Rajamuthiah et al., 2015). Niclosamide had activities against clinical isolates of vancomycin‐resistant *Enterococcus faecium* (VRE) and was superior to linezolid as a gastrointestinal decolonizing agent of vancomycin‐resistant *Enterococcus faecium*‐challenged mice (Mohammad et al., 2018). In this vancomycin‐resistant *Enterococcus faecium*‐colonization‐reduction mouse model, niclosamide (10 mg·kg^−1^·day^−1^ for 8 days, oral gavage) was administered to mice that were sensitized with ampicillin followed by *E. faecium* infection to establish gastrointestinal colonization. Tam et al. (2018) showed that niclosamide inhibited the pathogenesis of multidrug‐resistant *Clostridium difficile* by targeting entry of its toxins into colonocytes. In a mouse model of primary and recurrent *C. difficile* infection, post‐infection treatment with niclosamide (50 mg·kg^−1^·day^−1^ for 4 days, p.o.) ethanolamine (5% in DMSO) reduced both the primary disease and recurrence, without disrupting the gut microbiota (Tam et al., 2018). Niclosamide is stable in acidic pH and synergizes with metronidazole and proton pump inhibitors to eliminate *Helicobacter pylori* adhesion/invasion via multiple mechanisms such as reducing trans‐membrane pH, inhibiting IL‐8 (CXCL8) secretion and disrupting *H. pylori* proton motility (Tharmalingam et al., 2018). A screen of FDA‐approved drugs identified niclosamide as an inhibitor of the *Pseudomonas aeruginosa* quorum sensing signalling molecules (Imperi et al., 2013). Finally, niclosamide has significant activity against multidrug‐resistant *Mycobacterium tuberculosis* strains (Sun & Zhang, 1999) and inhibited its growth in infected human macrophages in a bacteriostatic manner (Fan et al., 2019). The same study also showed that it inhibited HIV replication in human macrophages via transcriptional effects.

## 6 NICLOSAMIDE AS AN ANTIVIRAL AGENT

Niclosamide role in anti‐viral host defence was first reported by Jurgeit et al. (2010) by the use of a monoclonal antibody against viral dsRNA during image‐based screening of infected cells. Niclosamide was shown to neutralize acidic membrane bound compartments via a proton carrier mode of action (protonophore) in vesicles as well as in protein‐free liposomes (Jurgeit et al., 2012). Blockade of the acidification of the endolysosomal compartments, without affecting vacuolar ATPase, has been shown to inhibit infection with the human rhinovirus and influenza virus in a pH‐dependent manner. The same mechanism was shown to mediate its antiviral efficacy against both Dengue and Zika viruses (Jung et al., 2019). A study found that niclosamide antiviral activity against Dengue virus was through a reduction of endosomal acidification and phosphorylation of AKT and p70SK (independent of mTOR) and against Zika virus through endosomal acidification and blocking the NS2B–NS3 interaction, thus highlighting its pleotropic antiviral effects (Kao et al., 2018; Z. Li et al., 2017). In a murine model of Dengue‐virus infection (suckling mice injected intracranially and i.p. with Dengue virus), treatment with niclosamide (5 mg·kg^−1^, i.p.) resulted in a significant reduction of viral replication in the brain and decreased mortality. However, exposure of niclosamide in the target tissues was not determined (Kao et al., 2018). Similarly, systemic administration of 1% niclosamide solution (50 mg·kg^−1^) to a humanized Zika‐virus infection model (embryonic chicks developing microcephaly) significantly reduced viral replication and restored cranial width and height (Cairns et al., 2018). Using a high‐throughput screening system and a subsequent entry assay, Wang et al. (2016) identified niclosamide as an inhibitor of virus entry, viral release and cell‐to‐cell transmission against Chikungunya virus and two other alphaviruses, Sindbis virus and Semliki forest virus. Li et al. (2017) found that niclosamide is a broad‐spectrum inhibitor against flaviviruses and also inhibited the replication of Ebola and Chikungunya viruses via the modulation of low pH‐dependent cellular mechanisms of viral maturation (Madrid et al., 2015; Mazzon et al., 2019). A systematic screen of FDA‐approved drugs identified niclosamide as one of the most potent Ebola virus inhibitors, although its *in vivo* efficacy is yet to be confirmed in animal models (Madrid et al., 2015). Finally, niclosamide also inhibits the pathogenic Beta‐CoVs (Gassen et al., 2019; Wen et al., 2007; Wu et al., 2004; Yang et al., 2020) and reduced the replication of Middle East respiratory syndrome coronaviruses (MERS‐Co‐V) via a mechanism involving enhanced autophagy through inhibition of S‐phase kinase‐associated protein 2 (SKP2) (Gassen et al., 2019).

## 7 NICLOSAMIDE AS A POTENT ANTI‐SARS‐CoV‐2 AGENT

Given niclosamide potent antiviral activity within the beta‐CoV family, it became apparent that it could be a potent antiviral against SARS‐CoV‐2. A study by Jeon et al. (2020) testing 3000 FDA‐approved drugs and other well‐characterized molecules identified niclosamide as the most potent inhibitor of SARS‐CoV‐2 in Vero cells, with a 40‐fold higher potency than remdesivir. Furthermore, Weiss et al. (2021) showed that niclosamide potency is conserved against the Alpha, Beta and Delta SARS‐CoV‐2 variant in Vero transmembrane serine protease 2 (TMPRSS2) cells and validated niclosamide strong antiviral activity in a human airway epithelial model. Niclosamide has also been shown to inhibit SARS‐CoV‐2 *in vivo*. Specifically, an inhaled niclosamide formulation was developed and tested in a murine infection model of SARS‐CoV‐2 (Brunaugh et al., 2021). Administration of niclosamide (0.24 mg·kg^−1^·day^−1^, intranasal) to SARS‐CoV‐2‐infected mice for 10 days improved survival and significantly reduced viral loads. Niclosamide further exhibited potent properties as an anti‐MRSA bacteriostatic agent and modulated various inflammatory cytokines such as IL‐1β, IL‐6 and TNF‐α. These findings suggest that niclosamide could also address secondary bacterial infections, which is one of the leading causes of death in COVID‐19 patients. It is evident that local administration in these viral infection models allowed substantially lower doses (0.25 and 5 mg·kg^−1^) compared with oral doses, thereby increasing the therapeutic window in terms of safety issues arising from excessive systemic exposure from oral dosing.

### 7.1 Mechanism of action against SARS‐CoV‐2

**FIGURE 2:**
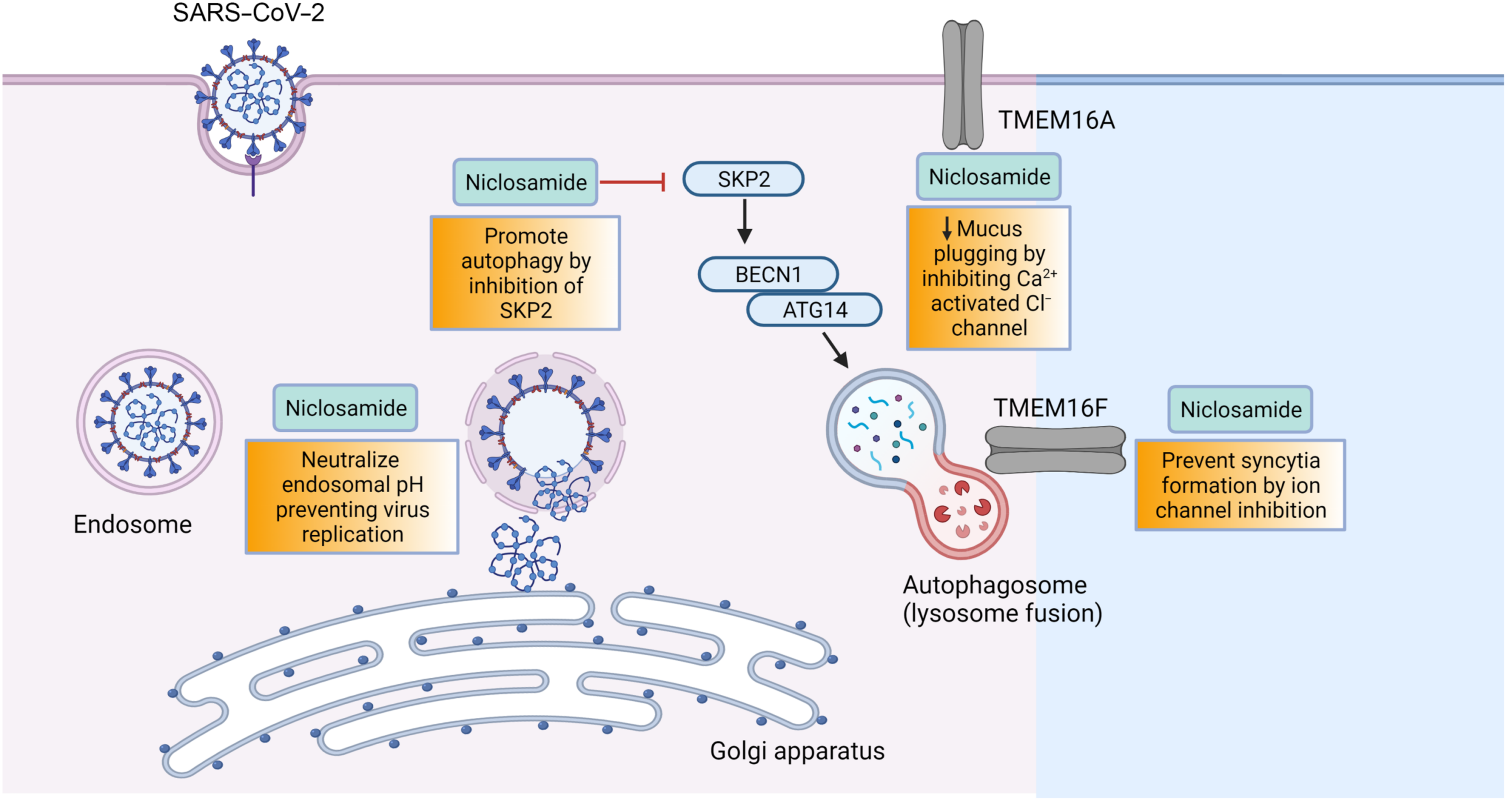
Potential mechanisms of antiviral activity have been illustrated in this figure. These include (i) endosomal pH neutralization to prevent viral replication, (ii) promotion of autophagy via inhibition of S‐phase kinase‐associated protein 2 (SKP2), (iii) decreased mucus plugging via inhibition of TMEM16A (calcium activated chloride channel; CaCC) and (iv) prevention of syncytia formation by ion channel inhibition. Abbreviation: BCN1, beclin‐1; ATG14, autophagy related 14; SARS‐COV‐2, severe acute respiratory syndrome coronavirus‐2. Created with BioRender.com

The antiviral activity of niclosamide against SARS‐CoV‐2 is complex and involves multiple cellular processes as illustrated in Figure 2. SARS‐CoV‐2 uses the angiotensin‐converting enzyme 2 (ACE2) as a cellular entry receptor in permissive cells of the respiratory tract and the spike proteins initiate the merging of the viral envelope with the host cell cytomembrane (Zhou et al., 2020). Following receptor binding and conformational changes in the spike protein, cathepsin L mediates proteolysis within endosomes leading to viral entry into host cells, as it has been shown for SARS‐CoV (Gomes et al., 2020; Huang et al., 2006; Simmons et al., 2005). The protonophoric activity of niclosamide that causes endosomal neutralization is thought to interfere with viral entry and egress preventing SARS‐CoV‐2 genome release and maturation. This mechanism has been derived from studies with pH‐dependent respiratory viruses—rhinovirus and influenza virus—which also use pH‐sensitive mechanisms of the endosomal pathway for viral entry and egress, similar to SARS‐CoV‐2 (Jurgeit et al., 2012). Garrett et al. (2021) recently demonstrated that the total lipid profile is amplified during SARS‐CoV‐2 infection in VeroE6 cells and treatment with niclosamide led to a reduction in lipids available for virus production. Additionally, in primary human lung cells and intestinal organoids, niclosamide enhances autophagy through inhibition of S‐phase kinase‐associated protein 2 thus further attenuating SARS‐CoV‐2 replication (Gassen et al., 2021).

Syncytia formation in SARS‐CoV‐2‐infected pneumocytes has been observed in COVID‐19 lungs. To identify inhibitors of spike‐driven syncytia formation, a high‐content microscopy‐based screening of more than 3000 compounds was conducted (L. Braga et al., 2021). The screen identified efficacious drugs that inhibited viral replication, with one of the most potent being niclosamide. Niclosamide also has potent bronchodilatory effects, inhibits excessive mucus production and down‐regulates the release of pro‐inflammatory cytokines such as IL‐8 by inhibiting TMEM16A (Cabrita et al., 2019). Due to its effects on intracellular calcium levels, niclosamide can inhibit other cytokines and could therefore play an important role in controlling the cytokine storm and acute respiratory distress syndrome (ARDS) in acutely ill COVID‐19 patients.

The above studies show several plausible mechanisms of action of niclosamide against COVID‐19, including prevention of viral entry, prevention of viral replication via autophagy inhibition and, finally, inhibition of spike‐driven syncytia formation. Importantly, unlike vaccines or monoclonal antibodies, the host‐directed mechanism of action of niclosamide means its antiviral efficacy is less likely to be affected by the emergence of SARS‐CoV‐2 variants, as has been shown for the Alpha, Beta and Delta variant by Weiss et al. (2021). In conclusion, these studies have confirmed potent, multi‐faceted and pleiotropic activity of niclosamide against SARS‐CoV‐2, targeting multiple aspects of the viral life cycle.

## 8 CLINICAL TRIALS OF NICLOSAMIDE FOR COVID‐19

**FIGURE 3:**
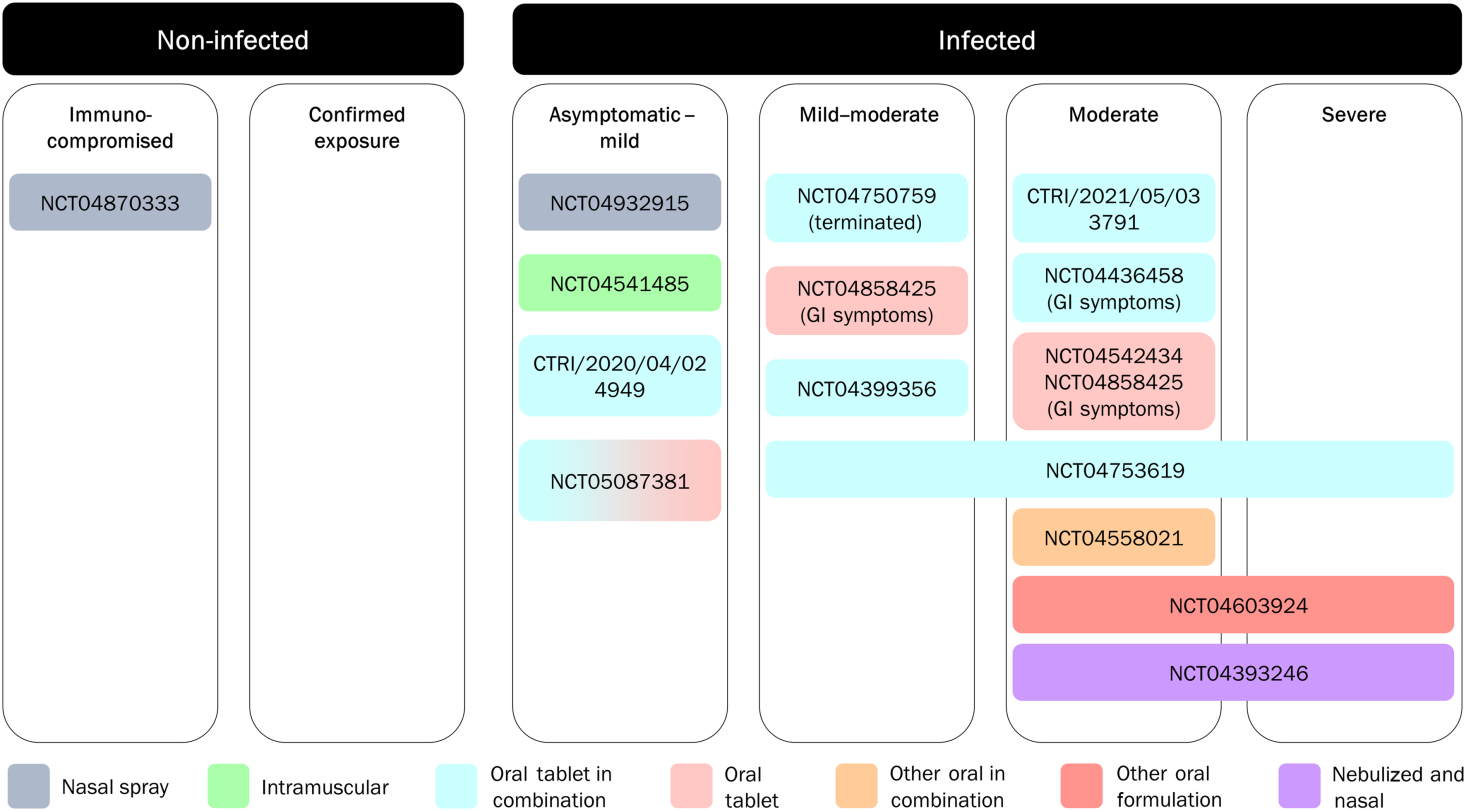
COVID‐19 clinical trials investigating niclosamide. Study references refer to National ClinicalTrials.gov (NCT), Clinical Trials Registry – India (CTRI) or EU Clinical Trials Register (EUCTR), where applicable, are included in Table 2. The majority of studies are dual listed on the WHO International Clinical Trials Registry Platform (ICTRP). Study NCT04372082 was terminated prior to randomization and is therefore excluded from the figure

Based on promising preclinical data, niclosamide is currently being evaluated as a potential COVID‐19 treatment in 18 human clinical trials relying on different formulations and/or routes of administration. Details of these trials can be found on public registries such as ClinicalTrials.gov and the WHO's Trial search website. Twelve of these trials are in Phase 2/3 and investigate the efficacy of niclosamide across the full COVID‐19 disease spectrum (Figure 3 and Table 2).

**TABLE 2:** COVID‐19 clinical trials utilizing niclosamide

Trial	Sample size and population	Intervention	Formulation and route of administration	Trial sponsor	Trial recruitment status
NCT04592835 Phase 1 to assess safety, tolerability and PK	24 healthy volunteers	DWRX2003 (niclosamide i.m.) versus placebo single ascending dose study	i.m.	Daewoong Pharmaceutical Co	^a^Not yet recruiting
NCT04524052 Phase 1 to assess safety, tolerability and PK/PD	32 healthy volunteers	DWRX2003 (niclosamide i.m. depot) versus placebo	i.m.	Daewoong Pharmaceutical Co	Not yet recruiting
NCT04749173 Phase 1 to assess safety, tolerability and PK	24 healthy volunteers	DWRX2003 (niclosamide i.m.) versus placebo	i.m.	Daewoong Pharmaceutical Co	Recruiting
NCT04541485 Phase 1 to assess safety, tolerability and PD	40 COVID‐19 patients (low–moderate risk)	DWRX2003 (niclosamide i.m. depot) versus placebo	i.m.	Daewoong Pharmaceutical Co	Not yet recruiting
NCT04576312 Phase 1	64 healthy volunteers	UNI911 (niclosamide inhalation + nasal spray) versus placebo	Solution for inhalation and intranasal application	UNION therapeutics	Completed, results published Backer et al. (2021)
NCT04644705 Phase 1 to assess safety and PK	28 healthy volunteers	Niclosamide and camostat versus placebo	p.o. tablet and novel galenic preparation	Charité Research Organisation GmbH	Recruiting
NCT04750759 EUCTR2020‐002233‐15‐DE Phase 2	40 participants with mild–moderate COVID‐19	Niclosamide and camostat tablet versus placebo	p.o. tablet	Charité Research Organisation GmbH	Terminated (subtherapeutic niclosamide plasma levels)
NCT04932915 EUCTR2021‐001036‐25‐DE Phase 2 study to assess safety and efficacy	330 asymptomatic or mild COVID‐19 participants	UNI91103 (niclosamide nasal spray) versus placebo	Solution for intranasal application	UNION therapeutics	Not yet recruiting
NCT04753619 Phase 2 open‐label randomized controlled trial	150 patients with COVID‐19 ranging from mild to severe	Niclosamide add on to standard of care	p.o. tablet	University of Baghdad	Completed, results published Abdulamir et al. (2021)
NCT04399356 Phase 2	73 participants with mild–moderate COVID‐19	Niclosamide + SoC versus placebo	p.o. tablet	Tufts Medical Center	Active, not recruiting
NCT04858425 Phase 2	148 participants with COVID‐19 gastrointestinal infection	Niclosamide versus placebo	p.o. tablet	AzurRx BioPharma	Recruiting
NCT04436458 Phase 2	100 participants with moderate COVID‐19 with gastrointestinal signs symptoms	Niclosamide + SoC versus placebo	p.o. tablet	First Wave Bio	Not yet recruiting
NCT04542434 Phase 2	148 participants with moderate COVID‐19 with gastrointestinal signs symptoms	Niclosamide versus placebo	p.o. tablet	First Wave Bio	Not yet recruiting
CTRI/2020/04/024949 Phase 2	48 participants with very mild COVID‐19	Niclosamide add on to standard of care	p.o. tablet	Lady Hardinge Medical College	Recruiting
CTRI/2021/05/033791 Phase 2	96 participants with moderate COVID‐19	Niclosamide + SoC versus SOC	p.o. tablet	Laxai Life Sciences Pvt Ltd	Recruiting
NCT04870333 EUCTR2020‐004144‐28‐GB Phase 2/3 Chemoprophylaxis trial in vulnerable patients	1500 participants from vulnerable patient populations	UNI91103 (niclosamide nasal spray) versus placebo	Solution for intranasal application	Cambridge University Hospitals NHS Foundation Trust	Recruiting
NCT04603924 Phase 2/3	436 participants with moderate–severe COVID‐19	ANA001 (niclosamide capsules) versus placebo	p.o. capsules	NeuroBo Pharmaceuticals	Recruiting
TACTIC‐E/NCT04393246 Phase 2/3	~469 participants with moderate–severe COVID‐19 (pre‐ICU)	EDP1815, dapagliflozin, ambrisentan, niclosamide, SoC	Solution for nebulization and intranasal application	Cambridge University Hospitals NHS Foundation Trust	Recruiting
NCT04558021 Phase 3	200 patients with moderate COVID‐19	Novel niclosamide suspension formulation as add‐on to SoC versus placebo	p.o. suspension	Imuneks Farma ilac San	Recruiting
NCT04372082 Phase 3	0	SoC versus SoC + hydroxychloroquine versus SoC + diltiazem–niclosamide	p.o. tablet	University Hospital, Lille	Withdrawn due to non‐effectiveness of hydroxychloroquine
NCT05087381 Phase 4	1800 participants with mild COVID‐19	SoC versus fluvoxamine versus fluvoxamine + bromhexine versus fluvoxamine + cyproheptadine versus niclosamide versus niclosamide + bromhexine	p.o. tablet	Chulalongkorn University	Recruiting

*Note*: Study references refer to National ClinicalTrials.gov (NCT), Clinical Trials Registry – India (CTRI) or EU Clinical Trials Register (EUCTR). The majority of studies are dual listed on the WHO International Clinical Trials Registry Platform (ICTRP).

^a^ A similar trial set‐up in the Philippines was terminated (9 December 2020) due to a lack of COVID‐19 patients (NCT04541485).

Abbreviations: PD, pharmacodynamics; PK, pharmacokinetics; SoC, standard of care.

In the pre‐symptomatic and mild COVID‐19 disease stage, the potent antiviral activity of niclosamide is thought to limit disease symptomatology and progression. However, as COVID‐19 progresses towards moderate and severe disease, the bronchodilatory and anti‐inflammatory effects of niclosamide might contribute to efficacy. Its antibacterial efficacy could also benefit COVID‐19 patients at risk for secondary bacterial infections, which is one of the leading causes of mortality in COVID‐19 (Cevik et al., 2020), particularly since the introduction of immunomodulators such as dexamethasone and tocilizumab as standard of care (SoC) medications.

### 8.1 Oral formulations

Of the 14 Phase 2/3 trials (Table 2), 11 trials are investigating oral formulations of niclosamide with seven using the marketed oral tablet form of niclosamide (also known under the tradename Yomesan), one utilizing capsules and one a novel suspension. However, one of these is registered as terminated due to subtherapeutic plasma levels and another one was withdrawn due to evidence showing the non‐effectiveness of hydroxychloroquine, which was being tested in one of the study arms.

Niclosamide in a chewable tablet form is being investigated in moderate COVID‐19 patients with gastrointestinal signs and symptoms at a dose of 400 mg three times per day for 14 days (National ClinicalTrials.gov (NCT) number 04542434, NCT04858425). Mortality, adverse event rate, faecal virus clearance and several clinical features are listed as outcome measures. Niclosamide in a chewable tablet form is also being investigated (in addition to standard of care(SoC) in asymptomatic/mild outpatient cases at 500 mg two times per day for 7–14 days (Clinical Trials Registry – India (CTRI)/2020/04/024949), mild–moderate cases at 2 g four times per day for 7 days (NCT04399356), moderate COVID‐19 subjects at 2 g·day^−1^ for 7 days (Clinical Trials Registry – India/2021/05/033791), moderately ill hospitalized COVID‐19 cases with gastrointestinal symptoms (NCT04436458) and mild–severe patients receiving a 2 g loading dose + 1 g every 12 h on Day 1 and then 1 g three times per day for 7 days (NCT04753619). The last trial was recently published (Abdulamir et al., 2021). This randomized, open‐label, controlled trial included 75 mild–severe COVID‐19 patients treated with SoC plus niclosamide (tablet) orally versus 75 patients receiving SoC only. Each group consisted of 25 mild, 25 moderate and 25 severe cases (defined according to WHO classification criteria). In moderate and severe, but not mild COVID‐19, patients receiving ‘niclosamide + SoC’ had a significantly shorter time to recovery, especially in patients with comorbidities compared with SoC only. Survival was not significantly increased. The small sample size in each disease severity group and the open‐label nature of the study have limited any robust conclusions being drawn from the study.

A niclosamide suspension is currently being tested in moderate hospitalized COVID‐19 patients receiving 200 mg (10 ml) niclosamide three times per day for 5 days (in addition to SoC), with time from admission to clinical recovery as the primary outcome measure. Another trial utilizes four 250 mg niclosamide capsules two times per day for seven consecutive days in moderate–severe cases defining safety and median time to hospital discharge as the primary outcome measures. Efficacy readouts of the other clinical trials investigating oral niclosamide preparations across the full COVID‐19 disease spectra are awaited.

### 8.2 Intramuscular injections

To address concerns around poor oral bioavailability of niclosamide, a formulation administered via the intramuscular route was developed. It is currently being investigated in healthy volunteers to assess its safety, tolerability and pharmacokinetics/pharmacodynamics (PK/PD) in 40 COVID‐19 patients (Table 2). This trial is a multiple‐dose ascending study injecting different volumes of a 24% suspension at four predefined injection sites. Incidence of treatment‐emergent adverse events and time to and rate of eradication of SARS‐CoV‐2 are the primary and secondary endpoints, respectively. Notably, Choi et al. (2021) performed a pharmacokinetics study in rats comparing equal doses of niclosamide administered via the intramuscular, intravenous and oral routes. They found increased systemic exposure with the intramuscular injection with a 70‐fold higher *C*_max_ and 13‐fold higher area under the curve (AUC) compared with the oral route. The intramuscular bioavailability was found to be 65% compared with 5.5% via the oral route, highlighting the substantial improvement via the intramuscular route.

### 8.3 Inhalational and intranasal administration

In another approach, the poor oral availability of niclosamide has been circumvented by developing a formulation optimized for inhalation and intranasal administration, aiming to achieve a high concentration in the lung (as the target tissue) whilst limiting systemic exposure to diminish side effects. Backer et al. (2021) published a randomized, double‐blind, placebo‐controlled Phase 1 trial assessing the safety and pharmacokinetics following inhaled (nebulized) and intranasal administration of a new formulation of niclosamide in healthy volunteers. Participants were randomly assigned to ascending single doses and five repetitive doses over 2.5 days. Inclusion criteria included a forced expiratory volume in 1 s (FEV1) of 80%. The study did not record any serious adverse events except mild irritation of the upper airways, increased fractional exhaled nitric oxide (FeNO) in 14.7% and an asymptomatic drop in forced expiratory volume in 1 s in 11.8% of subjects. A limitation of the study, however, was the exclusion of patients with underlying respiratory conditions such as asthma or chronic obstructive pulmonary disease (COPD), thus excluding patients who would be at the highest risk for adverse events via the inhalational route. The mean *C*_max_ of niclosamide following the highest dose of 52.9 mg was 337 ng·ml^−1^ (two times per day, repeat dosing) and 424 ng·ml^−1^ (single dose). This is lower compared with plasma levels of the (much higher) 2 g, p.o., dose used in anti‐helminthic treatment (Andrews et al., 1982; Burock, Daum, Keilholz, et al., 2018; Schweizer et al., 2018a, 2018b). The lower *C*_max_ would potentially limit the systemic side effects seen with oral dosing, a route that has been in use since the 1960s.

Following this Phase 1 study, three Phase 2/3 clinical trials were initiated, two of which are investigating the efficacy of the intranasal administration only—PROTECT‐V trial (PROphylaxis for paTiEnts at risk of COVID‐19 infecTion), overseen by the NIHR Covid‐19 Understanding and Elimination‐Trials Implementation Panel (CUE‐TIP) is a pre‐exposure prophylaxis trial in 1500 vulnerable renal patients who receive 140 μl of a 1% niclosamide ethanolamine solution (equivalent to 1.4 mg of niclosamide ethanolamine salt) in each nostril two times per day for up to 9 months, and PREVENT trial (asymptomatic/mild COVID‐19 patients receiving UNI91103 two times per day for 10 days). Finally, the TACTIC‐E trial (mulTi‐Arm therapeutiC sTudy in pre‐Icu patients admitted with COVID‐19—Experimental drugs) utilizes a combination of intranasal and nebulized niclosamide in moderate to severe COVID‐19 (Fisk et al., 2021; Lu et al., 2020; TACTIC‐E Trial, 2020). For the latter, combined intranasal and intra‐pulmonary (via the nebulized route) administration of niclosamide has the potential to be an efficacious approach as aerosol application of niclosamide via the inhaled and intranasal routes enables local delivery at the site of disease. Using this local delivery approach, niclosamide levels in target tissues are expected to be higher than plasma levels (Backer et al., 2021; UNION therapeutics, 2020) and achieve therapeutic concentrations at the primary site of infection. The targeted nasal administration is crucial because the nasopharynx and nasal cavity are both an entry point and a reservoir for SARS‐CoV‐2 (Gallo et al., 2021; Sungnak et al., 2020).

Overall, the breadth and spectrum of clinical trials utilizing niclosamide across the different disease stages of COVID‐19 will provide valuable human clinical and pharmacological data and have the potential to enable the development of niclosamide as an effective anti‐COVID‐19 agent, either in its own right or an as adjunct.

## 9 LIMITATIONS AND CHALLENGES TO THE CLINICAL USE OF NICLOSAMIDE

The favourable safety profile of niclosamide in treating humans with tapeworm infection could be due to the fact that the organ of interest was the gut and the drug did not need to be absorbed systemically and therefore did not get the chance to negatively modulate systemic signalling cascades. Systemic delivery is required for infections, cancers and metabolic diseases that have shown to be responsive to niclosamide. The safety profile of niclosamide in these conditions is largely unexplored and future studies are required for a clearer picture of its toxicity. The oral dose of niclosamide as a cestocidal agent is 2 g as a single dose, and this leads to a wide range of serum concentrations as described above (Andrews et al., 1982), mainly due to variable absorption rates. The combination of a low oral bioavailability and a wide range of serum concentrations results in unpredictable efficacy in clinical studies. Additional studies with a formulation that gives high bioavailability are required before niclosamide can be used more widely. One of the obstacles in this direction is that a direct target of niclosamide remains undiscovered.

There is great interest in conducting studies to elucidate the structure–activity relationship of niclosamide and thereby to identify novel derivatives of niclosamide that might have better bioavailability (H. Chen et al., 2013). A recent study that combined structure‐assisted drug design identified a mechanism‐based inhibitor (N3) and then determined the crystal structure of M^pro^ (main protease of SARS‐CoV‐2) in complex with this inhibitor (Z. Jin et al., 2020). Through a combination of structure‐based virtual and high‐throughput screening, the investigators assayed more than 10,000 compounds as inhibitors of M^pro^ and demonstrated that a robust screening strategy can rapidly discover drugs for new infections. Although these crystal structures provide useful new insights into drug discovery, extensive efforts are still needed to identify effective binding pockets for small molecules such as niclosamide and thereby validate the drug targets.

The challenges involved in repurposing niclosamide begin with its stable crystalline structure and its lipophilicity that restrict its solubility in water. This resulted in high oral doses in preclinical trials and therefore raised safety concerns for clinical trials as it made therapeutically relevant concentrations of the drug difficult to achieve. For example, in a Phase 1 dose‐escalation study testing oral niclosamide plus standard dose enzalutamide for prostate cancer, subjects on the higher dose (1000 mg three times per day) experienced dose‐limiting toxicities related to the gastrointestinal tract (colitis, diarrhoea, nausea and vomiting) resulting in the treatment being discontinued. However, plasma concentrations at the maximum tolerated dose (500 mg three times per day) were not consistently above the expected therapeutic threshold (Schweizer et al., 2018a, 2018b). The adverse events relating to the two patients who discontinued treatment (in the 1000 mg three times per day group) were most likely driven by the high local niclosamide concentrations in the gastrointestinal tract rather than the high systemic exposure. Peak plasma levels for the 1000 mg three times per day dose (*C*_max_ = 149–182 ng·ml^−1^, AUC_0 − *t*_ = 629–676 ng·h^−1^·ml^−1^ [min–max]) were below or similar to the plasma levels of studies using a 1.5 or 2 g daily dose, which is well tolerated (Burock, Daum, Tröger, et al., 2018). Of note, the COVID‐19 trials with niclosamide are using the approved 2 g p.o. daily dose (or lower inhalation/intranasal and intramuscular doses), and therefore, one would expect even smaller proportions of the drug to reach the gastrointestinal tract.

Improvement in pharmacological and pharmacokinetics properties through reformulation can help overcome some hurdles and make use of the drug more mainstream. In a Phase Ib prostate cancer trial, a novel reformulated orally bioavailable niclosamide/PDMX1001 (1200 mg three times per day) achieved plasma levels exceeding the therapeutic threshold (*C*_max_ = 70–236 ng·ml^−1^ and pre‐dose trough concentration *(C*_trough_)= 100–212 ng·ml^−1^ [min–max] vs. target dose of 32 ng·ml^−1^) when combined with abiraterone and prednisolone and was well tolerated with no dose‐limiting toxicities (Parikh et al., 2021). Zeyada et al. (2020) employed a novel oral niclosamide pluronic‐based nanoformulation and tested its effect in hepatocellular carcinoma in rats (70 mg·kg^−1^ 6 days·week^−1^ for 3 weeks, p.o.). These nanoparticles had sustained release properties up to 7 days and restored liver integrity, reduced alpha‐fetoprotein (AFP) levels and showed better anticancer activities compared with the drug alone. Furthermore, the trials described above using intramuscular injection or novel formulations of oral niclosamide in COVID‐19 will further elucidate its safety and efficacy, driven by systemic exposures.

Direct delivery of the drug into the respiratory and nasal routes could overcome some such hurdles and generate high drug concentrations at the site of primary infection in COVID‐19 infection, primarily the nasal cavity and lung tissue. Furthermore, this approach is thought to limit systemic exposure and hence decrease the risk of systemic side effects.

## 10 CONCLUSIONS AND FUTURE DIRECTIONS

Evidence has accumulated that niclosamide is a multi‐functional drug that can modulate several signalling pathways and biological processes. It has shown preclinical activity in many disease models, from cancer and metabolic diseases to various infections. The leading causes of mortality in COVID‐19 patients are an exaggerated immune response, as well as secondary bacterial infections and the development of acute respiratory distress syndrome. Niclosamide can function both as an anti‐bacteriostatic agent and as an immunomodulator; thus, it has unique advantages over other agents currently being tested in the COVID‐19 arena.

More importantly, niclosamide broad‐spectrum antiviral properties and potent inhibition of SARS‐CoV‐2 mean it can be developed rapidly as a cost‐effective therapeutic approach against COVID‐19 and hold the promise of widespread utilization as a primary or adjunctive agent. Niclosamide use could be further extended to other viral respiratory infections with a high unmet medical need, such as rhinovirus, influenza virus and respiratory syncytial virus. The reformulation of niclosamide into a nebulized and nasal route has the potential to provide the drug at therapeutic concentrations to the site of viral replication and disease and thereby minimize systemic toxicity. We anticipate that the results of the upcoming clinical trials of niclosamide in COVID‐19 will prove to be an important milestone in managing the pandemic globally.

### 10.1 Nomenclature of targets and ligands

Key protein targets and ligands in this article are hyperlinked to corresponding entries in the IUPHAR/BPS Guide to PHARMACOLOGY http://www.guidetopharmacology.org and are permanently archived in the Concise Guide to PHARMACOLOGY 2021/22 (Alexander, Christopoulos, Davenport, Kelly, Mathie, Peters, Veale, Armstrong, Faccenda, Harding, Pawson, Southan, Davies, et al., 2021; Alexander, Fabbro, Kelly, Mathie, Peters, Veale, Armstrong, Faccenda, Harding, Pawson, Southan, Davies, Beuve, et al., 2021a; Alexander, Fabbro, Kelly, Mathie, Peters, Veale, Armstrong, Faccenda, Harding, Pawson, Southan, Davies, Boison, et al., 2021b).

## AUTHOR CONTRIBUTIONS

JC devised the idea for the article. SS performed the initial literature search. SS, MF, JG, SK and JG wrote the first draft. AW designed Figure 1, IL designed Figure 2 and SS, AW, JG and JC designed Figure 3. SS, AW, JG, MS, RS and JC critically revised the article. All authors approved the final version.

## CONFLICT OF INTEREST

JC and RS acknowledge institutional grants from Union Therapeutics for the conduct of investigator initiated clinical trials of niclosamide. MS is a shareholder of UNION Therapeutics, and AW benefits from an employee incentive scheme. The other authors declare no conflict of interest.
